# E-cadherin relates to EGFR expression and lymph node metastasis in primary breast carcinoma.

**DOI:** 10.1038/bjc.1996.522

**Published:** 1996-10

**Authors:** J. L. Jones, J. E. Royall, R. A. Walker

**Affiliations:** Department of Pathology, University of Leicester, Glenfield Hospital, UK.

## Abstract

**Images:**


British Journal of Cancer (1996) 74, 1237-1241
© 1996 Stockton Press All rights reserved 0007-0920/96 $12.00

### E-cadherin relates to EGFR expression and lymph node metastasis in primary breast carcinoma

JL Jones, JE Royall and RA Walker

*Breast Cancer, Research Unit, Department of Pathology, University of Leicester, The Glenfield Hospital, Groby Road, Leicester LE3 9QP, UK.*

**Summary** Expression of the calcium-dependent cell-cell adhesion molecule E-cadherin has been examined in 187 primary breast carcinomas using an immunohistochemical technique. The pattern and extent of reactivity has been correlated with clinicopathological data including tumour type, grade and lymph node status and with other prognostic parameters including oestrogen receptor (ER) status, expression of c-*erb*B-2, pS2 protein and epidermal growth factor receptor (EGFR). Two patterns of E-cadherin staining were observed in carcinomas, membrane reactivity and a diffuse cytoplasmic staining. A marked difference in expression of E-cadherin was observed between infiltrating lobular carcinomas (ILC) and infiltrating ductal carcinomas (IDC), the former showing complete loss of membrane staining, whereas 93% of IDC retained some level of expression. In IDC reactivity was not related to tumour grade but there was a significant association between reduced membrane levels of E-cadherin and the presence of lymph node metastasis, and a highly significant correlation between the presence of cytoplasmic E-cadherin and metastasis. A significant relationship was also demonstrated between reduced E-cadherin reactivity and expression of EGFR. These findings emphasise the complexity of control of E-cadherin in breast carcinomas and provide evidence of a link between membrane signalling pathways and modulation of E-cadherin expression.

**Keywords:** breast carcinoma; E-cadherin; cytoplasmic reactivity; lymph node metastasis; epidermal growth factor receptor expression

Tumour cell invasion and metastasis is a multistep process requiring complex alterations in adhesive interactions. Detachment of tumour cells from the primary lesion is considered to be an important early step in the metastatic process (Coman, 1947). Adhesion is mediated by several families of cell adhesion molecules, including integrins (Hynes, 1992) and members of the immunoglobulin family (Springer, 1990). However, in epithelial cells E-cadherin (also known as L-CAM, uvomorulin and Arc-1) plays a critical role in initiating and maintaining cell-cell contacts (Takeichi, 1988).

E-cadherin is a member of the growing family of calcium-dependent cadherin adhesion molecules (Kemler, 1992). E-cadherin molecules are located within adherens junctions and are transmembrane structures mediating homotypic interactions between one or more of the extracellular cadherin repeats. The cytoplasmic region interacts with a group of proteins named the catenins, which in turn are connected to the actin microfilament network (Kemler, 1993).

That loss of E-cadherin function may be involved in tumour cell invasion has been demonstrated by an inverse relationship between expression of E-cadherin and invasive behaviour in some tumour cell lines (Frixen *et al.*, 1991), and this tumour-suppressor function has been supported by inhibition of tumour cell invasion following transfection of cells with E-cadherin cDNA (Vleminckx *et al.*, 1991). A more complex relationship has been observed between unstable or aberrant expression of cadherins and metastatic potential of tumour cells (Hashimoto *et al.*, 1989; Mareel *et al.*, 1991). While a clear role for E-cadherin in preventing tumour cell invasion has emerged from these *in vitro* studies, the relationship between E-cadherin expression in primary tumours and other prognostic parameters is inconsistent. In breast carcinomas, some studies have revealed a significant correlation between reduced E-cadherin expression and the presence of lymph node metastasis (Oka *et al.*, 1993), and with histological grade (Moll *et al.*, 1993). However, other studies have shown no such correlation (Lipponen *et al.*, 1994).

Evidence of the biological importance of E-cadherin in breast carcinomas is illustrated by the studies of D'Souza *et al.* (1994), who found that overexpression of *erb*B-2 in a non-tumorigenic mammary epithelial cell line leads to down-regulation of E-cadherin gene transcription. The *erb*B-2 proto-oncogene is a transmembrane tyrosine kinase receptor that shows homology to epidermal growth factor receptor (EGFR), and overexpression of this gene in breast carcinomas is associated with a rapid growth rate and a poor prognosis (Berger *et al.*, 1988; Walker *et al.*, 1989). That it also affects E-cadherin gene transcription suggests that this may be a mechanism whereby *erb*B-2 can affect the metastatic process.

A further relationship between the regulation of cell growth and E-cadherin expression is demonstrated by the culture of MCF-7 tumour cells in the presence of the anti-oestrogenic agent tamoxifen (Bracke *et al.*, 1994). This agent, which inhibits tumour cell growth, has been shown to restore E-cadherin-mediated adhesive function to these cells. Control of E-cadherin function can also be modulated by EGF. Kemler (1993) showed that *in vitro* activation of the EGFR with subsequent phosphorylation of its cytoplasmic domain is associated with dissociation of the E-cadherin/catenin complex from the cytoskeleton. Studies have shown that expression of EGFR by breast carcinomas is related to a poor prognosis (Sainsbury *et al.*, 1987) and Kemler's (1993) findings suggest that this may in part be mediated by altered E-cadherin function. However, in contrast to this, Oka *et al.* (1993) found a significant association between EGFR expression in primary breast carcinomas and retained E-cadherin reactivity.

Given this evidence of a relationship between E-cadherin function and growth control, as well as invasive behaviour *in vitro*, it is important to examine whether these associations are maintained in an *in vivo* situation. The aim of this study, therefore, was to examine the expression of E-cadherin in primary breast carcinomas and to relate this to clinicopathological parameters including tumour grade and lymph node status, and also to determine the relationship between E-

Correspondence: JL Jones
Received 2 November 1995; revised 16 April 1996; accepted 24 April 1996

cadherin reactivity and factors related to growth and poor prognosis, such as c-*erb*B-2 and EGFR expression, and to factors associated with less aggressive tumour behaviour such as oestrogen receptor (ER) positivity and expression of the oestrogen-regulated protein, pS2.

### Materials and methods

*Tissue specimens*

Fresh tumour tissue was collected from patients undergoing surgery between 1991 and 1994 and samples were immediately frozen in liquid nitrogen. Separate tissue was fixed in formol saline and paraffin embedded for routine histopathological assessment. A total of 187 specimens were examined including 156 infiltrating ductal carcinomas, 13 tubular carcinomas, 12 infiltrating lobular carcinomas and six cases of ductal carcinoma *in situ* (DCIS) with no invasive component. Histological grading of the tumours was carried out (RAW) using a modification of the Bloom and Richardson method (Elston *et al*., 1991). Information on lymph node status was known for 154 cases.

*Immunohistochemistry*

For detection of E-cadherin, 5 $\mu$m cryostat sections were cut onto silane-coated slides and allowed to air dry for 30 min. Sections were then fixed in acetone for 10 min at 4°C. The sections were incubated in normal rabbit serum (Sigma) for 10 min to block non-specific binding and then incubated overnight at 4°C with the mouse monoclonal anti-E-cadherin antibody (Euro-Path Ltd) at a dilution of 1:50 in Tris-buffered saline with 0.1% bovine serum albumin (BSA). A standard streptavidin–biotin complex (ABC) indirect immunoperoxidase technique was used to localise the bound antibody, which involved sequential incubation with biotinylated rabbit anti-mouse IgG at a dilution of 1:200 and streptavidin combined *in vitro* with biotinylated horseradish peroxidase (Dako), the reaction product being visualised using diaminobenzidine (DAB) hydrochloride.

Known positive cases (benign breast tissue) and negative controls (omission of the primary antibody) were included in each run and were shown to be positive and negative respectively. Normal breast epithelial elements in the tumour sections were used as internal positive controls.

Frozen tissue was also used for detection of oestrogen receptor (ER), using the ERICA Kit (Abbott), and for detection of EGFR, using EGFR1 monoclonal antibody (Amersham) at a dilution of 1:50. Formalin-fixed, paraffin-embedded tissue was used for detection of c-*erb*B-2, using the monoclonal antibody NCL-CB11 (Novocastra) at a dilution of 1:100, and for localisation of pS2 protein using the monoclonal antibody Histo CIS pS2 undiluted. In each case a standard ABC technique was used as described above. Known positive cases and negative controls were included in each run.

*Evaluation*

The extent and distribution of reactivity for E-cadherin was recorded in each case using a semi-quantitive scoring system. Staining was classed as 4 if >80% of the tumour showed reactivity, 3 if 50–80% was positive, 2 if 20–50% of the tumour showed staining, 1 for patchy, focal reactivity and 0 if there was no evidence of staining. This system was applied to each tumour to determine the extent of reactivity for each of the two patterns of localisation observed, membrane and cytoplasmic.

Tumours were recorded as ER positive if >10% of the tumour cells showed staining and, similarly, sections stained for pS2 were considered positive if >10% of the tumour was staining. Reactivity for c-*erb*B-2 and EGFR was also recorded.

E-cadherin expression was compared with tumour type and grade, lymph node status in 154 cases, ER status in 161 cases, EGFR reactivity in 159 cases, c-*erb*B-2 expression in 110 cases and pS2 expression in 102 cases.

*Statistics*

For statistical analysis, the chi-squared test was used for correlation with all pathological data.

### Results

*E-cadherin reactivity in relation to clinicopathological data*

Cases of normal and benign breast tissue (fibrocystic change and fibroadenoma) were used as positive controls for E-cadherin reactivity. These demonstrated strong membrane staining of the glandular epithelial cells with localisation at the intercellular borders (Figure 1). Non-epithelial cells did not express E-cadherin. Normal breast epithelial elements within tumour sections displayed similar strong reactivity.

In contrast to normal breast tissue, two patterns of staining for E-cadherin were observed in carcinomas, with reactivity either localised at the cell membrane in a similar manner to that seen in normal and benign epithelium, or distributed diffusely within the cytoplasm, a pattern not observed in non-malignant epithelium. Of the 156 infiltrating ductal carcinomas (IDC), nine showed no evidence of staining for E-cadherin, either membrane or cytoplasmic. The remaining 147 IDC showed variable levels of membrane staining for E-cadherin, although in no case was this as strong as the level of reactivity observed in normal glandular elements (Figure 2). In addition to membrane staining, in many of the tumours a variable proportion of cells exhibited cytoplasmic reactivity for E-cadherin, in some cases this being the predominant pattern observed (Figure 3). Of the 13 cases of tubular carcinoma examined, one showed no reactivity for E-cadherin, either membrane or cytoplasmic, despite a normal distribution of E-cadherin in adjacent non-malignant ducts. The other tubular carcinomas displayed variable levels of membrane and cytoplasmic staining in a pattern similar to that of the IDC.

In marked contrast to the IDC, none of the 12 cases of infiltrating lobular carcinomas included in the study showed any evidence of membrane reactivity for E-cadherin (Figure 4), although in three cases there was weak cytoplasmic reactivity in a small proportion of the tumour cells. Three of the cases also contained areas of lobular carcinoma *in situ* (LCIS), which were similarly negative for E-cadherin.

The six cases of pure DCIS, including one case of comedo-

**Figure 1** Section of normal breast tissue showing strong membrane staining for E-cadherin in epithelial cells. There is no staining of non-epithelial elements.

type DCIS, all exhibited normal or near normal membrane expression of E-cadherin. No significant cytoplasmic reactivity was observed.

The extent and pattern of staining for E-cadherin was compared with the tumour grade of the IDC. Of the 156 cases, 24 were histological grade I, 61 grade II and 71 grade III. While there was a trend towards the better differentiated tumours retaining membrane reactivity for E-cadherin, this did not reach statistical significance. There was no relationship between tumour grade and the presence of cytoplasmic E-cadherin (Table I). The absence of reactivity observed in nine IDC was also not related to tumour grade. The 13 tubular carcinomas, all by definition grade I, displayed variable levels and patterns of staining, some having predominantly cytoplasmic reactivity, others with near normal levels of membrane staining.

When the pattern of E-cadherin expression was correlated with the lymph node status of the patient, both for the IDC alone and the IDC together with the tubular carcinomas, there was a significant association between reduced membrane reactivity and the presence of lymph node metastasis ($0.005 > P > 0.001$). Interestingly, a highly significant association was demonstrated between the presence of cytoplasmic reactivity for E-cadherin in the tumour cells and lymph node metastasis ($P < 0.001$) (Table I), including two unusual cases of tubular carcinoma which showed extensive cytoplasmic reactivity. The lack of membrane E-cadherin in the infiltrating lobular carcinomas did not relate to lymph node metastasis, with eight cases being lymph node-positive and two being lymph node-negative. Similarly, there was no relationship to the presence of cytoplasmic reactivity.

*E-cadherin reactivity in relation to other markers*

For the infiltrating ductal carcinomas, no significant correlation was observed between the extent and pattern of E-cadherin reactivity and ER status, c-*erb*B-2 expression or pS2 expression (Table I). However, a statistically significant relationship was evident between the presence of cytoplasmic reactivity for E-cadherin in the tumour cells and EGFR positivity ($0.01 > P > 0.005$). This relationship could also be demonstrated at the cellular level, with colocalisation of the cytoplasmic E-cadherin and EGFR within tumour groups. No such relationship was seen for the presence of membrane reactivity and EGFR positivity. These relationships were maintained when the tubular carcinomas were included in this group.

The groups of infiltrating lobular carcinoma and DCIS were too small for statistical analysis. However, no clear relationship with any of the factors was evident.

**Figure 2** Moderately differentiated infiltrating ductal carcinoma displaying membrane reactivity for E-cadherin on the majority of tumour cells.

### Discussion

The process of tumour progression, invasion and metastasis is a complex cascade of events (Hart *et al.*, 1989), which involves escape from normal growth control mechanisms, invasion of surrounding stroma and release of neoplastic cells from the primary tumour with subsequent establishment and growth at a distant site. Many of these processes imply altered adhesive function of the neoplastic cells, and in epithelial tumours, altered E-cadherin function has been shown to play a critical role in allowing release of neoplastic cells from a normal cohesive structure. However, there is recent evidence that different stages of tumour development may be closely linked, with mitogenic signals also affecting cell adhesion (D'Souza *et al.*, 1994), and this study has evaluated whether these relationships are maintained *in vivo*.

A number of studies have established an inverse correlation between E-cadherin expression and tumour differentiation (Schipper *et al.*, 1991; Kinsella *et al.*, 1993). In infiltrating ductal carcinomas (IDC) of the breast, the relationship of E-cadherin expression to clinicopathological

**Figure 3** A moderately differentiated infiltrating ductal carcinoma displaying predominantly diffuse cytoplasmic reactivity for E-cadherin in the tumour cells.

**Figure 4** Absence of reactivity for E-cadherin in an infiltrating lobular carcinoma (T). The adjacent normal duct (N) displays strong membrane staining.

**Table I** Correlation of E-cadherin reactivity in infiltrating ductal carcinomas and tubular carcinomas with clinicopathological features and other markers

| | *Mem*<*50%* | *Mem*>*50%* | P-*value* | *Cyt*<50% | *Cyt*>*50%* | P-*value* |
|---|---|---|---|---|---|---|
| Grade I | 51% ($n$=19) | 49% ($n$=18) | | 54% ($n$=20) | 46% ($n$=17) | |
| Grade II | 70% ($n$=43) | 30% ($n$=18) | $\chi^2$=5.09 | 61% ($n$=37) | 39% ($n$=24) | $\chi^2$=3.25 |
| Grade III | 72% ($n$=51) | 28% ($n$=20) | NS | 45% ($n$=32) | 55% ($n$=39) | NS |
| LN positive | 78% ($n$=57) | 22% ($n$=16) | $\chi^2$=9.43 | 26% ($n$=19) | 74% ($n$=54) | $\chi^2$=39.3 |
| LN negative | 56% ($n$=45) | 44% ($n$=36) | 0.005>$P$>0.001 | 77% ($n$=62) | 23% ($n$=19) | <0.001 |
| c-*erb*B-2+ | 64% ($n$=18) | 36% ($n$=10) | $\chi^2$=1.6 | 43% ($n$=12) | 57% ($n$=16) | $\chi^2$=0.57 |
| c-*erb*B-2– | 77% ($n$=63) | 23% ($n$=19) | NS | 51% ($n$=42) | 49% ($n$=40) | NS |
| EGFR+ | 72% ($n$=28) | 28% ($n$=11) | $\chi^2$=0.76 | 36% ($n$=14) | 64% ($n$=25) | $\chi^2$=6.696 |
| EGFR– | 64% ($n$=77) | 36% ($n$=43) | NS | 59% ($n$=71) | 41% ($n$=49) | 0.01>$P$>0.005 |
| ER+ | 63% ($n$=69) | 37% ($n$=40) | $\chi^2$=1.13 | 54% ($n$=59) | 46% ($n$=50) | $\chi^2$=0.001 |
| ER– | 71% ($n$=37) | 29% ($n$=15) | NS | 54% ($n$=28) | 46% ($n$=42) | NS |
| pS2+ | 69% ($n$=50) | 31% ($n$=22) | $\chi^2$=0 | 50% ($n$=36) | 50% ($n$=36) | $\chi^2$=0.38 |
| pS2– | 67% ($n$=8) | 33% ($n$=4) | NS | 42% ($n$=5) | 58% ($n$=7) | NS |

Mem, membrane staining; cyt, cytoplasmic staining; LN, lymph node; NS, not significant.

parameters is inconsistent. Moll *et al.* (1993) and Oka *et al.* (1993) found that reduced E-cadherin related to poor differentiation. The latter group also demonstrated a significant association between reduced E-cadherin staining and the presence of lymph node metastasis. In contrast to this, Lipponen *et al.* (1994) examined 179 IDC and found no correlation with tumour grade or lymph node status. In our study 146/156 (93%) of IDC showed some evidence of staining, but, while there was a trend towards grade I IDC retaining E-cadherin expression, there was no statistically significant relationship between the extent or pattern of reactivity to tumour grade. However, in keeping with the observations of Oka *et al.* (1993), there was a significant correlation between reduced membrane staining for E-cadherin and a positive lymph node status. The presence of diffuse cytoplasmic reactivity for E-cadherin has previously been reported in certain tumours (Pignatelli *et al.*, 1994), although the significance of this distribution has not been addressed. We demonstrated a highly significant association between tumour cytoplasmic reactivity for E-cadherin and the presence of lymph node metastasis. There are a number of possible explanations for this finding. One is that the cytoplasmic reactivity indicates a reversible change in E-cadherin expression by the cell in response to the local environment. It has been demonstrated that E-cadherin expression can easily be altered *in vitro* in response to the culture environment (Mareel *et al.*, 1991) and that highly metastatic ovarian tumour cells have unstable E-cadherin expression (Hashimoto *et al.*, 1989). Such a temporal disruption of E-cadherin-mediated adhesion would allow tumour cell detachment, while re-expression could favour colonisation at a distant site. In support of this, there are reports of increased E-cadherin expression in metastatic lesions compared with their primary tumour (Mayer *et al.*, 1993).

Alternatively, such aberrant E-cadherin localisation may be the result of abnormal catenin expression. Incorporation of E-cadherin into stable cell–cell contacts is modulated by the cytoplasmic proteins, α-, β-and γ-catenin (Hirano *et al.*, 1992; Hinck *et al.*, 1994). Thus, loss of one or more of the catenin proteins may be expected to result in weak association of E-cadherin to the cytoskeleton, distribution diffusely within the cytoplasm and loss of adhesive function. Interestingly, altered expression of γ-catenin (plakoglobin) has recently been reported in human breast cancer cells (Sommers *et al.*, 1994). It would be of interest to examine the association of cytoplasmic reactivity for E-cadherin with catenin gene expression.

There is increasing evidence that E-cadherin function may be modulated by growth factor activity. Hoschuetzky *et al.* (1994) have demonstrated the physical association of EGFR with E-cadherin via β-catenin, while Shiozaki *et al.* (1995) have shown that phosphorylation of EGFR leads to dissociation of E-cadherin/catenin complexes from the cytoskeleton. In keeping with this, our study demonstrates a significant correlation between the expression of EGFR by tumour cells and the presence of cytoplasmic reactivity for E-cadherin. This may illustrate one mechanism whereby local environmental influences could modulate both tumour cell growth and invasive behaviour. Oka *et al.* (1993) have reported an association between EGFR positivity and retained E-cadherin expression by tumours, although the localisation of this reactivity was not reported.

Further evidence linking cadherins to signalling pathways is illustrated by the down-regulation of E-cadherin transcription in response to overexpression of the tyrosine kinase receptor *erb*B-2 in a non-tumorigenic human mammary epithelial cell line (D'Souza *et al.*, 1994). Our study showed no relationship between c-*erb*B-2 expression and E-cadherin reactivity, demonstrating perhaps the complexity of factors involved in tumour progression.

Possession of ER and reactivity for the oestrogen-regulated protein, pS2, are generally indicative of tumours with a good prognosis (Foekens *et al.*, 1990). Transfection of a mouse mammary adenocarcinoma cell line with pS2 cDNA has been shown to alter cell phenotype, giving the previously cohesive cell colonies a more dispersed structure (Williams *et al.*, 1995) and suggesting altered adhesive interactions, although our study does not support a direct effect on E-cadherin expression. In contrast to Lipponen *et al.* (1994), we found no correlation between E-cadherin expression and ER status.

While E-cadherin reactivity was maintained in 93% of infiltrating ductal carcinomas, none of the infiltrating lobular carcinomas or LCIS showed evidence of membrane E-cadherin. These findings are in keeping with those of other workers (Moll *et al.*, 1993; Lipponen *et al.*, 1994) and suggest distinct modes of invasion in these two cancer types. Interestingly, a similar pattern of staining is seen in diffuse-type gastric carcinomas, which are also characterised by lack of glandular differentiation and single, non-polarised cells infiltrating stroma (Mayer *et al.*, 1993). Furthermore, mutations in the E-cadherin gene have been discovered in this form of gastric carcinoma (Becker *et al.*, 1994). In infiltrating lobular carcinoma of breast also, loss of mRNA for E-cadherin has been demonstrated (Rasbridge *et al.*, 1995), and in some cases gene mutations have been identified (Kanai *et al.*, 1994; Berx *et al.*, 1995).

In conclusion, the role of E-cadherin in breast tumour progression appears increasingly complex. Whereas in ILC loss of E-cadherin appears to be an early event in tumour development, in IDC altered E-cadherin expression appears to be more directly related to the metastatic process. The association of cytoplasmic E-cadherin with lymph node metastasis suggests a sophisticated control over cell–cell adhesion possibly in response to local growth factors such as EGF. This link between E-cadherin expression and signalling pathways may be an important stage in understanding tumour progression and examination of the dynamic interaction between these two factors, in particular the control of catenin expression in relation to this, merits further study.

**Acknowledgements**

JL Jones is supported by the Royal College of Pathologists BUPA Research Fellowship. J Royall is supported by the Cancer Research Campaign.

### References

BECKER K-F, ATKINSON MJ, REICH U, BECKER I, NEKARDA H, SIEWERT JR AND HOFLER H. (1994). E-Cadherin gene mutations provide clues to diffuse type gastric carcinomas. *Cancer Res.*, **54**, 3845–3852.

BERGER MS, LOCHER GW, SAURER S, GULLICK WJ, WATERFIELD MD, GRONER B AND HYNES NE. (1988). Correlation of c-*erb*B-2 gene amplification and protein expression in human breast carcinoma with nodal status and nuclear grading. *Cancer Res.*, **48**, 1238–1243.

BERX G, CLETON-JANSEN A-M, NOLLET F, DE LEEUW JF, VAN DE VIJVER MJ, CORNELISSE C AND VAN ROY F. (1995). E-Cadherin is a tumour/invasion suppressor gene mutated in human lobular breast cancers. *EMBO J.*, **14**, 6107–6115.

BRACKE ME, CHARLIER C, BRUYNEEL EA, LABIT C, MAREEL MM AND CASTRONOVO V. (1994). Tamoxifen restores the E-Cadherin function in human breast cancer MCF 7/6 cells and suppresses their invasive phenotype. *Cancer Res.*, **54**, 4607–4609.

COMAN DR. (1947). Mechanisms of the invasiveness of cancer. *Science*, **105**, 347–348.

D'SOUZA B AND TAYLOR-PAPADIMITRIOU J. (1994). Overexpression of ERBB2 in human mammary epithelial cells signals inhibition of transcription of the E-Cadherin gene. *Proc. Natl Acad. Sci. USA*, **91**, 7202–7206.

ELSTON CW AND ELLIS IO. (1991). Pathological prognostic factors in breast cancer I. The value of histological grade in breast cancer: experience from a large study with long term follow up. *Histopathology*, **19**, 403–410.

FOEKENS JA, RIO M-C, SEGUIN P, VAN PUTTEN WL, FAUQUE J, NAP, M, KILJN JG AND CHAMBON P. (1990). Prediction of relapse and survival in breast cancer patients by pS2 protein status. *Cancer Res.*, **50**, 3832–3837.

FRIXEN UH, BEHRENS J, SACHS M, EBERLE G, VOSS B, WARDA A, LOCHNER D, AND BIRCHMEIER W. (1991). E-Cadherin mediated cell–cell adhesion prevents invasiveness of human carcinoma cells. *J. Cell Biol.*, **113**, 173–185.

HART IR, GOODE NT AND WILSON RE. (1989). Molecular aspects of the metastatic cascade. *Biochim. Biophys. Acta*, **989**, 65–84.

HASHIMOTO M, NIWA O, NITTA Y, TAKEICHI M AND YOKORO K. (1989). Unstable expression of E-Cadherin adhesion molecules in metastatic ovarian tumour cells. *Jpn. J. Cancer Res.*, **80**, 459–463.

HINCK L, NATHKE IS, PAPKOFF J AND NELSON WJ. (1994). Dynamics of cadherin/catenin complex formation: novel protein interactions and pathways of complex assembly. *J. Cell Biol.*, **125**, 1327–1340.

HIRANO S, KIMOTO N, SHIMOYAMA Y, HIROHASHI S AND TAKEICHI M. (1992). Identification of a neural α-catenin as a key regulator of cadherin function and multicellular organisation. *Cell*, **70**, 293–301.

HOSCHUETZKY H, ABERLE H AND KEMLER R. (1994). β catenin mediates interaction of the cadherin–catenin complex with epidermal growth factor receptor. *J. Cell Biol.*, **127**, 1375–1380.

HYNES R. (1992). Integrins: versatility, modulation and signaling in cell adhesion. *Cell*, **69**, 11–25.

KANAI Y, ODA T, TSUDA H, OCHIAI A AND HIROHASHI S. (1994). Point mutation of the E-Cadherin gene in invasive lobular carcinoma of the breast. *Jpn. J. Cancer Res.*, **85**, 1035–1039.

KEMLER R. (1992). Classical cadherins. *Semin. Cell Biol.*, **3**, 149–155.

KEMLER R. (1993). From cadherins to catenins: cytoplasmic protein interactions and regulation of cell adhesion. *Trends Genet.*, **9**, 317–321.

KINSELLA AR, GREEN B, LEPTS GC, HILL CL, BOWIE G AND TAYLOR BA. (1993). The role of cell adhesion molecule E-cadherin in large bowel tumour cell invasion and metastasis. *Br. J. Cancer*, **67**, 904–909.

LIPPONEN P, SAARELAINEN E, JI. H, AALTOMAA S AND SYRJANEN K. (1994). Expression of E-cadherin (E-Cd) as related to other prognostic factors and survival in breast cancer. *J. Pathol.*, **174**, 101–109.

MAREEL MM, BEHRENS J, BIRCHMEIER W, DE BRYNE GK, VLEMINCKX K, HOOGEWIJS A, FIERS WC AND VAN ROY FM. (1991). Down regulation of E-cadherin expression in Madin Darby canine kidney (MDCK) cells inside tumours of nude mice. *Int. J. Cancer*, **47**, 922–928.

MAYER B, JOHNSON JP, LEITL F, JAUNCH KW, HEISS MM, SCHILDBERG FW, BIRCHMEIER W AND FUNKE I. (1993). E-cadherin expression in primary and metastatic gastric cancer: down regulation correlates with cellular dedifferentiation and glandular disintegration. *Cancer Res.*, **53**, 1690–1695.

MOLL R, MITZE M, FRIXEN UH, BIRCHMEIER W. (1993). Differential loss of E-cadherin in infiltrating ductal and lobular breast carcinomas. *Am. J. Pathol.*, **143**, 1731–1742.

OKA H, SHIOZAKI H, KOBAYASHI K, INOVE M, TAHARA H, KOBAYASHI T, TAKATSUKA Y, MATSUYOSHI N, HIRNO S, TAKEICHI M AND MORI T. (1993). Expression of E-cadherin cell adhesion molecules in human breast cancer tissues and its relationship to metastasis. *Cancer Res.*, **53**, 1696–1701.

PIGNATELLI M, ANSARI TW, GUNTER P, LIU D, HIRANO S, TAKEICHI M, KLOPPEL G AND LEMOINE NR. (1994). Loss of membranous E-cadherin expression in pancreatic cancer: correlation with lymph node metastasis, high grade and advanced stage. *J. Pathol.*, **174**, 243–248.

RASBRIDGE SA, POULSOM R, MILLIS RR. (1995). *In-situ* hybridisation for E-cadherin in breast carcinoma. *J. Pathol.*, **175**, 142A.

SAINSBURY JRC, FARNDON JR, NEEDHAM GK, MALCOLM AJ AND HARRIS AL. (1987). Epidermal growth factor receptor status as a predictor of early recurrence of and death from breast cancer. *Lancet*, **8547**, 1398–1402.

SCHIPPER JH, FRIXEN UH, BEHRENS J, UNGER A, JAHNKE K AND BIRCHMEIER W. (1991). E-cadherin expression in squamous carcinomas of head and neck: inverse correlation with tumour dedifferentiation and lymph node metastasis. *Cancer Res.*, **51**, 6328–6337.

SHIOZAKI H, KADOWAKI T, DOKI Y, INOUE M, TAMURA S, OKA H, IWAZAWA T, MATSUI S, SHIMAYA K, TAKEICHI M AND MORI T. (1995). Effect of epidermal growth factor on E-cadherin mediated adhesion in a human oesophageal cancer cell line. *Br. J. Cancer*, **71**, 250–258.

SOMMERS CL, GELMANN EP, KEMLER R, COWIE P AND BYERS SW. (1994). Alterations in beta catenin phosphorylation and plakoglobin expression in human breast cancer cells. *Cancer Res.*, **54**, 3544–3552.

SPRINGER T. (1990). Adhesion receptors of the immune system. *Nature*, **346**, 425–434.

TAKEICHI M. (1988). The cadherins: cell–cell adhesion molecules controlling animal morphogenesis. *Development*, **102**, 639–655.

VLEMINCKX K, VAKAET L, MAREEL MM, FIERS W AND VAN ROY F. (1991). Genetic manipulation of E-cadherin expression by epithelial tumour cells reveals an invasion suppressor role. *Cell*, **66**, 107–119.

WALKER RA, GULLICK W AND VARLEY JM. (1989). An evaluation of immunoreactivity for c-*erb*B-2 protein as a marker of short term prognosis in breast cancer. *Br. J. Cancer*, **60**, 426–429.

WILLIAMS R, LALANI E-N, PIGNATELL M, PLAYFORD R AND STAMP GWH. (1995). Dispersed growth pattern of mouse mammary adenocarcinoma cell line 410.4 transfected with pS2 cDNA on 2D and 3D collagen gels. *J. Pathol.*, **175S**, 103A.


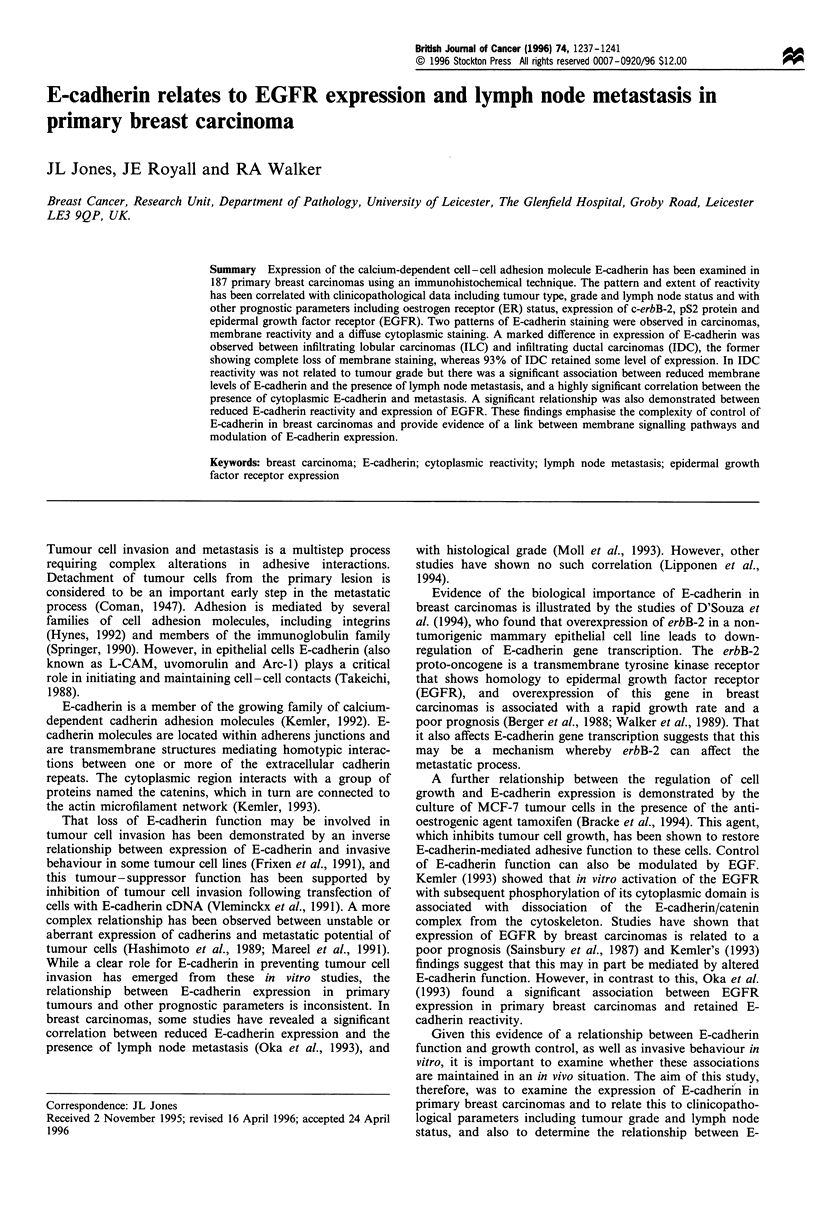

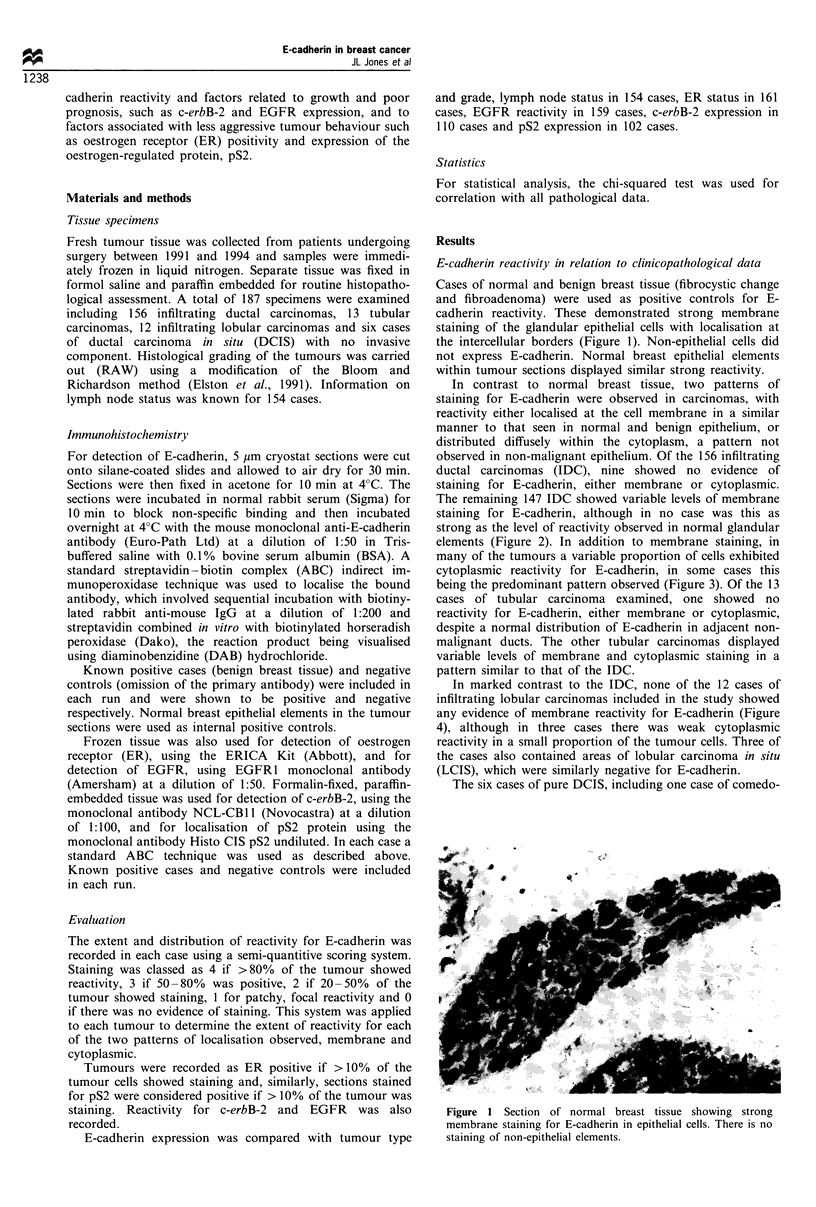

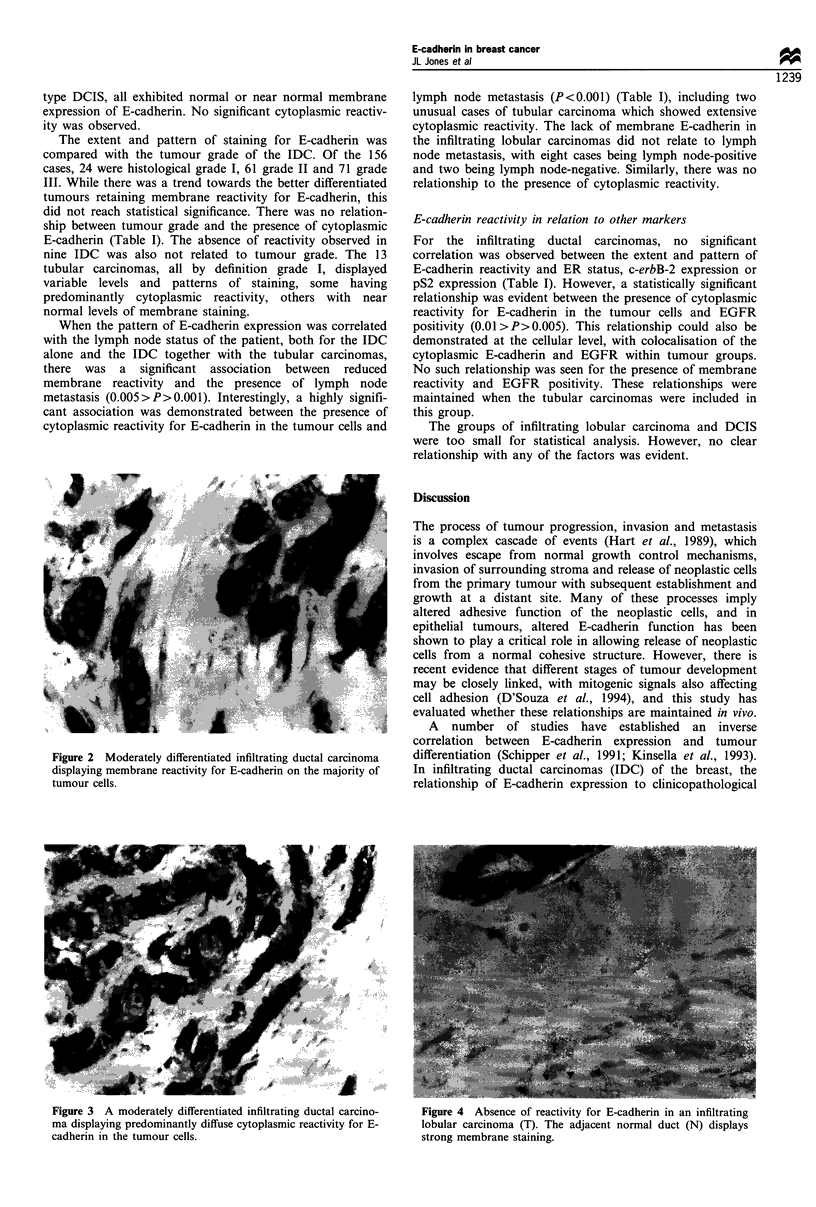

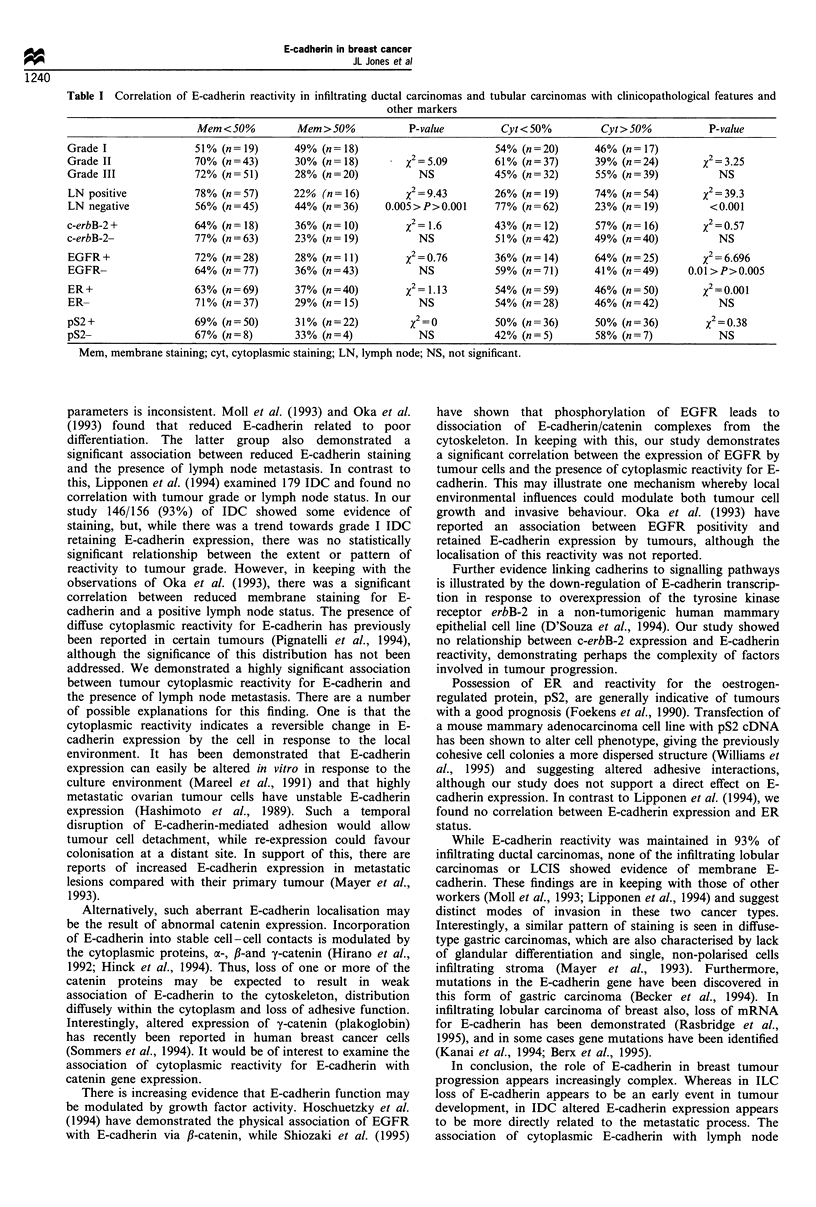

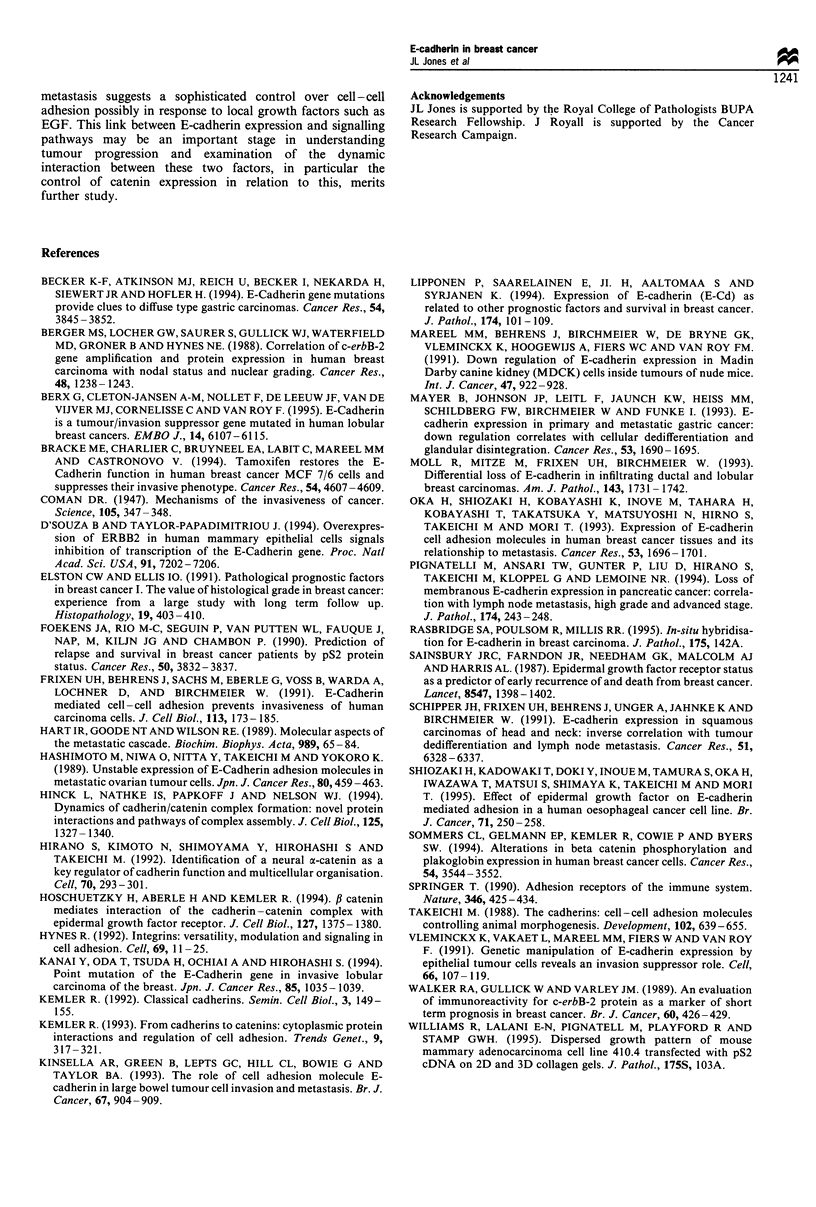


## References

[OCR_00520] Becker K. F., Atkinson M. J., Reich U., Becker I., Nekarda H., Siewert J. R., Höfler H. (1994). E-cadherin gene mutations provide clues to diffuse type gastric carcinomas.. Cancer Res.

[OCR_00526] Berger M. S., Locher G. W., Saurer S., Gullick W. J., Waterfield M. D., Groner B., Hynes N. E. (1988). Correlation of c-erbB-2 gene amplification and protein expression in human breast carcinoma with nodal status and nuclear grading.. Cancer Res.

[OCR_00533] Berx G., Cleton-Jansen A. M., Nollet F., de Leeuw W. J., van de Vijver M., Cornelisse C., van Roy F. (1995). E-cadherin is a tumour/invasion suppressor gene mutated in human lobular breast cancers.. EMBO J.

[OCR_00539] Bracke M. E., Charlier C., Bruyneel E. A., Labit C., Mareel M. M., Castronovo V. (1994). Tamoxifen restores the E-cadherin function in human breast cancer MCF-7/6 cells and suppresses their invasive phenotype.. Cancer Res.

[OCR_00541] Coman D. R. (1947). Mechanism of the Invasiveness of Cancer.. Science.

[OCR_00547] D'souza B., Taylor-Papadimitriou J. (1994). Overexpression of ERBB2 in human mammary epithelial cells signals inhibition of transcription of the E-cadherin gene.. Proc Natl Acad Sci U S A.

[OCR_00553] Elston C. W., Ellis I. O. (1991). Pathological prognostic factors in breast cancer. I. The value of histological grade in breast cancer: experience from a large study with long-term follow-up.. Histopathology.

[OCR_00557] Foekens J. A., Rio M. C., Seguin P., van Putten W. L., Fauque J., Nap M., Klijn J. G., Chambon P. (1990). Prediction of relapse and survival in breast cancer patients by pS2 protein status.. Cancer Res.

[OCR_00563] Frixen U. H., Behrens J., Sachs M., Eberle G., Voss B., Warda A., Löchner D., Birchmeier W. (1991). E-cadherin-mediated cell-cell adhesion prevents invasiveness of human carcinoma cells.. J Cell Biol.

[OCR_00569] Hart I. R., Goode N. T., Wilson R. E. (1989). Molecular aspects of the metastatic cascade.. Biochim Biophys Acta.

[OCR_00573] Hashimoto M., Niwa O., Nitta Y., Takeichi M., Yokoro K. (1989). Unstable expression of E-cadherin adhesion molecules in metastatic ovarian tumor cells.. Jpn J Cancer Res.

[OCR_00579] Hinck L., Näthke I. S., Papkoff J., Nelson W. J. (1994). Dynamics of cadherin/catenin complex formation: novel protein interactions and pathways of complex assembly.. J Cell Biol.

[OCR_00583] Hirano S., Kimoto N., Shimoyama Y., Hirohashi S., Takeichi M. (1992). Identification of a neural alpha-catenin as a key regulator of cadherin function and multicellular organization.. Cell.

[OCR_00591] Hoschuetzky H., Aberle H., Kemler R. (1994). Beta-catenin mediates the interaction of the cadherin-catenin complex with epidermal growth factor receptor.. J Cell Biol.

[OCR_00595] Hynes R. O. (1992). Integrins: versatility, modulation, and signaling in cell adhesion.. Cell.

[OCR_00597] Kanai Y., Oda T., Tsuda H., Ochiai A., Hirohashi S. (1994). Point mutation of the E-cadherin gene in invasive lobular carcinoma of the breast.. Jpn J Cancer Res.

[OCR_00604] Kemler R. (1992). Classical cadherins.. Semin Cell Biol.

[OCR_00608] Kemler R. (1993). From cadherins to catenins: cytoplasmic protein interactions and regulation of cell adhesion.. Trends Genet.

[OCR_00614] Kinsella A. R., Green B., Lepts G. C., Hill C. L., Bowie G., Taylor B. A. (1993). The role of the cell-cell adhesion molecule E-cadherin in large bowel tumour cell invasion and metastasis.. Br J Cancer.

[OCR_00620] Lipponen P., Saarelainen E., Ji H., Aaltomaa S., Syrjänen K. (1994). Expression of E-cadherin (E-CD) as related to other prognostic factors and survival in breast cancer.. J Pathol.

[OCR_00623] Mareel M. M., Behrens J., Birchmeier W., De Bruyne G. K., Vleminckx K., Hoogewijs A., Fiers W. C., Van Roy F. M. (1991). Down-regulation of E-cadherin expression in Madin Darby canine kidney (MDCK) cells inside tumors of nude mice.. Int J Cancer.

[OCR_00633] Mayer B., Johnson J. P., Leitl F., Jauch K. W., Heiss M. M., Schildberg F. W., Birchmeier W., Funke I. (1993). E-cadherin expression in primary and metastatic gastric cancer: down-regulation correlates with cellular dedifferentiation and glandular disintegration.. Cancer Res.

[OCR_00639] Moll R., Mitze M., Frixen U. H., Birchmeier W. (1993). Differential loss of E-cadherin expression in infiltrating ductal and lobular breast carcinomas.. Am J Pathol.

[OCR_00642] Oka H., Shiozaki H., Kobayashi K., Inoue M., Tahara H., Kobayashi T., Takatsuka Y., Matsuyoshi N., Hirano S., Takeichi M. (1993). Expression of E-cadherin cell adhesion molecules in human breast cancer tissues and its relationship to metastasis.. Cancer Res.

[OCR_00652] Pignatelli M., Ansari T. W., Gunter P., Liu D., Hirano S., Takeichi M., Klöppel G., Lemoine N. R. (1994). Loss of membranous E-cadherin expression in pancreatic cancer: correlation with lymph node metastasis, high grade, and advanced stage.. J Pathol.

[OCR_00663] Sainsbury J. R., Farndon J. R., Needham G. K., Malcolm A. J., Harris A. L. (1987). Epidermal-growth-factor receptor status as predictor of early recurrence of and death from breast cancer.. Lancet.

[OCR_00666] Schipper J. H., Frixen U. H., Behrens J., Unger A., Jahnke K., Birchmeier W. (1991). E-cadherin expression in squamous cell carcinomas of head and neck: inverse correlation with tumor dedifferentiation and lymph node metastasis.. Cancer Res.

[OCR_00673] Shiozaki H., Kadowaki T., Doki Y., Inoue M., Tamura S., Oka H., Iwazawa T., Matsui S., Shimaya K., Takeichi M. (1995). Effect of epidermal growth factor on cadherin-mediated adhesion in a human oesophageal cancer cell line.. Br J Cancer.

[OCR_00680] Sommers C. L., Gelmann E. P., Kemler R., Cowin P., Byers S. W. (1994). Alterations in beta-catenin phosphorylation and plakoglobin expression in human breast cancer cells.. Cancer Res.

[OCR_00686] Springer T. A. (1990). Adhesion receptors of the immune system.. Nature.

[OCR_00692] Takeichi M. (1988). The cadherins: cell-cell adhesion molecules controlling animal morphogenesis.. Development.

[OCR_00696] Vleminckx K., Vakaet L., Mareel M., Fiers W., van Roy F. (1991). Genetic manipulation of E-cadherin expression by epithelial tumor cells reveals an invasion suppressor role.. Cell.

[OCR_00700] Walker R. A., Gullick W. J., Varley J. M. (1989). An evaluation of immunoreactivity for c-erbB-2 protein as a marker of poor short-term prognosis in breast cancer.. Br J Cancer.

